# Chemical composition and insecticidal efficacy of two animals’ horn and hoof crude extracts against lesser grains borer, *Rhyzopertha dominica* (F.) [Coleoptera: Bostrichidae]

**DOI:** 10.1016/j.heliyon.2025.e41778

**Published:** 2025-01-08

**Authors:** Kayode David Ileke, Catherine Olukemi Adeniran

**Affiliations:** a Department of Biology, School Life of Sciences, Federal University of Technology, P. M. B. 704, Akure, Ondo State, Nigeria; b Department of Animal Production and Health, School of Agriculture and Agricultural Technology, Federal University of Technology,P. M. B. 704, Akure, Ondo State, Nigeria

**Keywords:** Wheat, Bioactive, Mortality, *Rhyzopertha dominica*, *Bos taurus*, *Capra hircus*

## Abstract

This research evaluated the profiling of bioactive compounds and insecticidal activities of the crude extracts of horns and hoofs of cow and goat against *Rhyzoperta dominica* on wheat grains. Different concentrations (0.1, 0.2, 0.3, 0.4 and 0.5 ml) of the crude extracts were applied per 20 g of wheat grains to assess the toxicity effect on adult mortality and adult emergence. The different concentrations of the two extracts evoked mortality of *R. dominica*. However, the most effective concentration was 0.5 ml of the crude extract of goat horn causing 100 % mortality after 5days of application, while extract of cow hoof at the same concentration resulted in 86.67 % mortality of *R. dominica* after 5 days of exposure. The required lethal concentration needed to control 50 % population of *R. dominica* by extract of cow horn and goat hoof after 24 h exposure was 0.57 ml and 0.48 ml, respectively. Proximate compositions of the animal hoofs and horns showed that the samples contained fat, crude protein and ash. The number of bioactive compounds detected in cow hoof was 44 and goat horn was 21. The study has shown the insecticidal potential of the understudied animal materials. The use of extracts from the horns of goats should be looked into and explored as an eco-friendly measure in the control of *R. dominica.*

## 1 Introduction

Wheat is one of the world's most essential staple crops which play an important role in ensuring global food security [1]. It serves as a primary food source for billions of people across the globe, forming the foundation of countless diets and providing sustenance to diverse populations. Wheat takes many forms and is a fundamental part of the daily meal for individuals from different cultural backgrounds [2].

Wheat is the second most widely grown crop globally, trailing only maize [2,3]. It is cultivated across a diverse range of climates, from the temperate regions of North America and Europe to the subtropical areas of Asia and Africa [4]. The sheer ubiquity of wheat cultivation makes it a dependable source of sustenance, contributing to food security by ensuring a consistent supply of this vital grain. Wheat is inherently nutritious, providing a valuable source of carbohydrate, dietary fiber, and essential nutrients such as vitamins and minerals. These attributes make wheat-based foods a staple source of energy for individuals worldwide [5]. The wheat industry represents a substantial portion of the global agricultural economy. It serves as a source of livelihood for millions of farmers, making it not only a contributor to food security but also an engine of economic stability [6].

Despite the numerous importance of wheat, it faces challenges, particularly when it comes to post-harvest preservation. One of the most insidious threats to wheat storage is the infestation by insect pests, and among these pests are *Rhyzopertha dominica*, *Sitophilus oryzae*, *S. zeamais*, *S. granarius*, *Tribolium castaneum*, *T. confusum*, *Sitotroga cerellela* and *Plodia interpunctella* [2,7] which stand out as formidable adversaries [8]. Insect pests pose a substantial threat to stored wheat, jeopardizing food security on multiple fronts. *Rhyzopertha dominica* infestation can cause up to 30 % weight loss in wheat grain in Africa [9]. In Egypt and Morocco, *R. dominica* infestation had been reported to caused 25 % [10] and 20 % weight loss [11] in wheat grains respectively. Estimated annual loss due to *R. dominica* infestation in wheat grain in Africa is about $300 million [9]. It had been reported in Egypt that an annual loss due to *R. dominica* infestation of wheat grain was $75 million [10]. *Rhyzopertha dominica* have a global presence and has shown a particular affinity for infesting wheat storage facilities [12]. Global annual losses of $1.5 billion to $2.5 billion due to *R. dominica* has been reported [13]. The estimation of these losses depends on several factors like location and storage conditions. This insect pest causes extensive damage to wheat grains by burrowing into them, consuming the nutritious kernel, and leaving behind a trail of destruction. The result is not only a significant reduction in grain quality but also a loss in quantity, which can have dire consequences for food security. In addition to physical damage, insect pests can introduce contaminant, such as feces and excretions, into the grain which does not only reduce the nutritional quality of the stored wheat but can also pose health risks to consumers [14]. If the insects are not properly managed, these infestations can have long-term consequences, leading to food losses and economic burdens for both individual farmers and the entire communities [15].

Stored Products Entomologists are faced with the challenges of development of an ecofriendly insecticides that will reduced side effects pose by synthetic chemical insecticide such as environmental health hazard [16]. The use of plant products had been exploited to control insect pests of stored cereals and legumes with some merits [17]. Some plants products have bioactive compounds that can act as defense against insect pests [18]. The use of animal wastes in insect pests control strategy is gaining more attention in some developing Africa countries [16,19,20]. Research work on the utilization of animal hoofs and horns is scarce in literature, most especially on its use in the control of *R. dominica* on wheat grains. Therefore, this research investigated the insecticidal activities of hoofs and horns of *Bos taurus* and *Capra hircus* against *Rhyzopertha dominica*. Proximate and mineral compositions of the hoofs and horns were also investigated.

## 2 Materials and methods

### 2.1 Insects rearing

Adult *Rhyzopherta dominica* were provided by the Department of Biology, Storage Entomology Research Laboratory, Federal University of Technology, Akure. One hundred pairs of the weevils were introduced to 1-L glass kilner jars containing 500 g of wheat (variety Sokoto) each, procured from a grain dealer in Akure. A consistent insectarium environment of 28 ± 2 °C and 75.5 % relative humidity was maintained for the insect colonies [2,16].

### 2.2 Collection and preparation of animal horns and hoofs for extraction

The horns and hoofs of cows were obtained from abattoir in Akure, Ondo State, Nigeria, while the horns and hoofs of West African Dwarf goats were obtained from butchers in Erekesan market in Akure, Ondo State and were transported to the laboratory. After being transported to a laboratory, the animal horns and hoofs were cleaned thoroughly with clean running water, allowed to air dry for four weeks, and then ground separately in a hammer mill. Before use, the powders were stored separately in labeled, airtight plastic containers and refrigerated at 4 °C.

Hoof and horn powder weighing 500g each were individually steeped in an extraction bottle holding 800 ml of pure ethanol. A glass rod was used to agitate the mixture every 6 h, and the extraction process was stopped after 72 h following the method described by Ileke et al. [21]. Whatman No. 1 filter papers were used in double layers for the filtration process, and the solvent was evaporated using a rotary evaporator set to run at 30–40 °C for 8 h at a speed of 3–6 rpm as described by Udo [22]. The crude extract was exposed at room temperature to remove residual ethanol.

### 2.3 Collection of wheat grains

The grains of wheat used for the research work were obtained from newly stocked wheat grains (variety Sokoto) free of insecticides from a grain merchant at the Erekesan market in Akure, Ondo State. The grains were cleaned by removing dirt, stones and broken kennels before sterilizing using a deep freezer maintained at -5 °C for 72 h to eliminate insect eggs, larvae, pupa and adult which might be present. The disinfected wheat grains were removed from the freezer, air dried in the laboratory for 3 h to prevent moulding [21,23].

### 2.4 Determination of proximate composition

The proximate analytical procedures were utilized to evaluate the protein, moisture, ash, and fat composition of the goat and cow hoofs and horns in this investigation [24].

### 2.5 Mineral content determination

Triple acid digestion method described by Pujar et al. [25] was employed to determine mineral content. Two (2) grams each of the animal material powder was weighed into a micro-kjeldhal digestion flask and 24 ml mixed concentrated Tetraoxosulphate (IV) acid (H_2_SO_4_), Nitric acid (HNO_3_) and 60 % Perchloric acid (HClO_4_) in ratio 9:2:1 v/v were introduced. A transparent solution was obtained by heating and digesting the mixture. The contents were cooled before being transferred to a 50 ml volumetric flask and filled to the appropriate level with water. The solution was used to determine the mineral elements such as iron (Fe), calcium (Ca), potassium (K), and sodium (Na). The iron (Fe) content was determined with UV/visible spectrophotometer of model PFP7. A flame photometer model RVC/06–08 was used to evaluate the presence of calcium (Ca), potassium (K), iron (Fe), zinc, manganese (Mn), and copper (Cu) in the samples.

### 2.6 Chemical compounds in the extracts

The chemical components found in the extract of horns and hoofs of cow and goat were identified using gas chromatography combined with mass spectrometry (GC-MS). Agilent Technologies was used to analyze 1 μL of each extract. The models of the machine used include: Mass Spectrum (5975C VLMSD), Injector (7683B Series) and GC (7890A) with helium as the carrier gas. The HP-5MS capillary column measured of 30 cm in length, 0.320 mm in internal diameter, and 0.25 μm in film thickness. The GC oven temperature was set at 80 °C for 2 min. The samples were allowed to run for 36 min, the temperature was gradually raised to 240 °C at a rate of 6 °C per minute. Each chemical compound's peak is expressed in terms of its balance and retention time.

### 2.7 Toxicity of animal horns and hoofs oil extracts on adult mortality and emergence

Cow and goat horns and hoofs ethanolic extracts were tested at concentrations of 0.1, 0.3, 0.4, and 0.5 ml/20 g of wheat grains put inside plastic container (250 ml). The treated wheat grain was shaken vigorously for 1 min to enhance proper coating of the grains. Ten pairs (10 males: 10 females) [26] of newly emerged (7 days old) adults of *R. dominica* were introduced into each of the treated plastic containers separately while the control experiment had only 20 g of wheat grains and ten copulating pair of *R. dominica*. The set-up was replicated three times and arranged out in Complete Randomized Design. The mortality rate of the insect was measured daily for five days. The dead weevils were the ones that neither moved nor responded to pin probing. Percentage adult mortality was corrected using Abbot [27] formula.

The insect bioassay setup was kept inside the insect rearing cage for thirty-five days for the first filial generation (F1) to emerge.

### 2.8 Data analysis

The mean and level of significance for the differences between means of the data acquired were determined using the New Duncan's Multiple Range Test at P < 0.05 after the data were subjected to one-way analysis of variance (ANOVA). Adulticidal bioassay data were analysed using the log-Probit model to determine the 50 % lethal concentration (LC_50_) and the 90 % lethal concentration (LC_90_).

## 3 Results

### 3.1 Proximate contents of cow and goat horns and hoofs

The proximate content from horn and hoof of cow and goat is presented in Table 1. The highest moisture content was recorded from goat hoof (12.70 %), while the lowest was recorded from cow hoof (8.33 %). There is no significant difference (p > 0.05) in the moisture content recorded for cow horn (9.92 %) and goat hoof (10.42 %). The fat content of the samples was different significantly (p < 0.05); cow horn has the highest fat content of 16.52 %, followed by goat horn (15.96 %). The lowest fat content of 13.43 % was recorded from cow hoof. Protein content in cow horn (59.92) was significantly higher (p < 0.05) compared to in cow hoof (57.18), goat horn (56.15) and goat hoof (57.57). Cow horn has the highest ash content of 10.81 %, which was significantly different (p < 0.05) compared to goat horn (10.08 %), goat hoof (6.67 %) and cow hoof (5.19 %). The fibre content in cow horn (1.46 %) was significantly higher (p < 0.05) compared to cow hoof (0.79 %), goat hoof (0.69 %) and goat horn (0.77 %).

**Table 1:** Proximate analysis of cow and goat horns and hoofs.

Animal horns and hoofs	Proximate content (%)				
	Moisture content	Fat	Protein	Ash	Fibre
CHN	9.92 ± 0.50b	16.52 ± 0.15d	59.92 ± 0.24b	10.81 ± 0.11d	1.46 ± 0.06b
CHF	8.33 ± 0.23a	13.43 ± 0.15a	57.18 ± 0.43a	5.19 ± 0.16a	0.79 ± 0.02a
GHN	12.70 ± 0.19c	15.96 ± 0.16c	56.15 ± 0.43a	10.08 ± 0.19c	0.77 ± 0.08a
GHF	10.42 ± 0.25b	14.92 ± 0.15b	57.57 ± 1.60a	6.67 ± 0.10b	0.69 ± 0.08a

Mean with the same letter in superscript along column are not significantly different from one another (*p* > 0.05) using Duncan Multiple Range Test.

Keys: CHN: cow horn; CHF: cow hoof; GHN: goat horn; GHF goat hoof.

### 3.2 Composition of trace elements in cow and goats horns and hoofs

The quantitative of trace elements in extracted oil from horn and hoof of cow and goat is represented in Table 2. Trace elements such as Na, K, Ca, Mn, Mg, Cu, Fe, Zn, and P were detected in varying quantity from horn and hoof of cow and goat. The quantity of Na present in animal samples were different significantly (p < 0.05); goat hoof has the highest amount of Na (82.13), followed by cow horn (75.27). The quantity of K and Ca present in goat hoof was 110.10 and 52.10, respectively. This amount was significantly higher (p < 0.05) compared to the amount in cow hoof, 95.13 and 47.47, respectively. The lowest amount K (84.63) and Ca (35.77) was observed in goat horn. Cow horn has the highest (4.02) amount of Mn, followed by goat hoof with 3.02. Cow hoof and goat horn have 2.84 and 2.99 of Mn, respectively. Cow hoof has the highest amount of Mg (41.29), this is significantly different (p < 0.05) from the amount observed from other oil; the lowest amount of Mg was recorded from goat hoof (34.57). The highest (1.53) amount of Cu was recorded from goat horn followed by cow hoof (1.24). Goat horn (2.10) was rich in Fe compared to goat hoof (2.03), cow hoof (1.84), and cow horn (1.58). The Zn content in goat hoof (4.02) is significantly higher (p < 0.05) compared to the goat horn (2.83), cow hoof (3.28) and cow horn (3.12). Phosphorous present in cow hoof is significantly higher (19.39), compared to the amount present in goat hoof (10.52). The lowest amount of P was recorded in goat horn oil (1.57).

**Table 2:** Trace element in cow and goat horns and hoofs.

Animal Oil	Trace Element								
	Na	K	Ca	Mn	Mg	Cu	Fe	Zn	P
CHN	75.27 ± 0.09c	92.47 ± 0.09b	43.13 ± 0.07b	4.02 ± 0.003d	35.63 ± 0.03b	1.05 ± 0.003b	1.58 ± 0.008a	3.12 ± 0.01b	3.84 ± 0.02b
CHF	69.45 ± 0.09a	95.13 ± 0.09c	47.47 ± 0.03c	2.84 ± 0.004a	41.29 ± 0.00d	1.24 ± 0.004c	1.84 ± 0.002b	3.28 ± 0.01c	19.39 ± 0.03d
GHN	70.86 ± 0.07b	84.63 ± 0.07a	35.77 ± 0.09a	2.99 ± 0.003b	39.13 ± 0.07c	1.53 ± 0.002d	2.10 ± 0.058c	2.83 ± 0.02a	1.57 ± 0.02a
GHF	82.13 ± 0.09d	110.10 ± 0.06b	52.10 ± 0.09d	3.02 ± 0.010c	34.57 ± 0.03a	0.87 ± 0.014a	2.03 ± 0.014c	4.02 ± 0.01d	10.52 ± 0.02c

Mean with the same letter in superscript along column are not significantly different from one another (*p* > 0.05) using Duncan Multiple Range Test.

**Keyes:** CHN: cow horn; CHF: cow hoof; GHN: goat horn; GHF goat hoof.

### 3.3 Chemical component of cow hoof oil

**Fig. 1:**
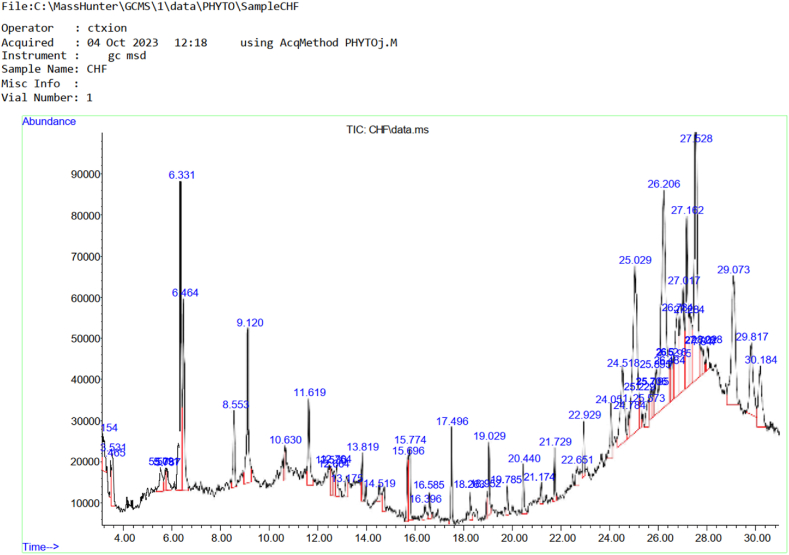
Chromatogram of the chemical compounds present in Cow hoof.

The chemical compounds found in cow hoof oil extract is presented in Table 3 and Chromatogram of the chemical compounds present in cow hoof is also presented in Fig. 1. Total number of 44 bioactive compounds were identified from the oil sample. The compound with the highest area percentage among the identified compounds was 1,2-Benzenediol,3,5-bis(1,1-dimethylethyl) with area percent of 10.79, this was followed by Undec-10-ynoic acid tetradecyl ester with 7.99 area percent and 1,2-Bis(trimethylsilyl)benzene with area percent of 5.81. Other notable compounds present include Cyclotrisiloxane (tert-butyldimethysilyloxy and terimethsilyl), 3-Hydromandelic acid,1H-Isonidole-1,3(2H)-dione,2-buty1-4,5,6,7-tetrahydro-, Indole-2-one,2,3-dihydro-N-hydroxy-4-methoxy-3,3-dimethyl-, and Arsenous acid tris (trimethylsilyl)ester.

**Table 3:** Chemical compounds present in cow hoof oil extract.

S/N	Compound Detected	Retention Time	Area %
1	Cyclotrisiloxane, Hexamethyl-1,2-bezisothiazol-3-amine (tert-butyldimethysilyloxy)	3.154	0.83
2	Cyclotrisiloxane, Hexamethyl-1,2-bezisothiazol-3-amine (terimethsilyl)	3.465	0.46
3	9H-Fluorene-2-carboxylicacid	3.531	1.06
4	Silicic acid	5.731	3.26
5	3-Hydromandelic acid	8.553	1.32
6	Cyclopentasiloxane, decamethl-cylopentasiloxane	9.120	3.22
7	1,3,5,7,9-Pentaethylcyclopentasiloxane	10.630	0.46
8	Cyclohexasiloxane, dodecamethyl-cyclohexasiloxane	11.619	1.86
9	Pyrido[-2,3-d]pyrimidine,4-phenyl-	12.530	0.52
10	Cyclotrisiloxane	12.764	0.60
11	Carvacol	13.175	0.57
12	Cyclopentasiloxane	13.819	0.60
13	5,5′-Di(ethoxycarbonyl)-3,3′-dimethyl-4,4′-dipropyl-2,2′-dipyrrylmethane	14.519	0.33
14	Cyclopentasiloxane, decamethyl-3,4-dihydroxyphenylglycol	15.696	0.99
15	Epineprine,(.beta.)-,3TMS derivative	15.774	1.47
16	Octasiloxane,1,1,3,3,5,5,7,7,9,9,11,11,13,13,15,15- hexadecamethyl-	17.496	1.27
17	1H-Indole-2-carboxylic acid,6-(4-ethoxyphenyl)-3-methyl-4-oxo-4,5,6,7-tetrahydro-,Isopropyl ester	18.263	1.43
18	Tris(tert-butyldimethylsilyloxy)ar sane	18.952	0.34
19	Heptasiloxane,1,1,3,3,5,5,7,7,9,9,11,11,13,13-tetradecamethyl-	19.029	1.03
20	2,4-Dihydroxybenzoic acid, 3TMS derivative	20.440	0.70
21	Arsenous acid, tris(trimethylsilyl)ester	21.174	0.36
22	4,7,7-Trimethylbicyclo[2.2.1]heptan-2-one 0-allylxime	22.651	0.57
23	1,2,5-Oxadiazole-3-amine,4-(4-methoxyphenoxy)-	22.929	0.69
24	1H-Indole-2-carboxylic acid	24.05	1.02
25	1,2-Benzenediol,3,5-bis(1,1-dimethylethyl)-	24.518	3.26
26	4H-1,2,4-triazole-3,5-diamine,N3-($-fluorophenyl)-N5-methyl-	24.784	0.50
27	Undec-10-ynoic acid, tetradecyl ester	25.029	7.99
28	2-methyl-z,z-3,13-octadecadienol	25.229	0.54
29	Pyrazolo[5,1-c][1,2,4]triazine-3,8-dicarboxylic acid,4-amino-,diethyl ester	25.573	0.75
30	11,13-Dimethyl-12-tetradecen-1-ol acetate	25.706	0.65
31	2-Myristynoyl-glycinamide	25.795	0.49
32	1H-Isonidole-1,3(2H)-dione,2-buty 1–4,5,6,tetrahydro-	25.895	1.19
33	1,2-Benzenediol,3,5-bis(1,1-dimethylethyl)-	26.206	10.79
34	Ethyl 2(-2-chloroacetamido)-3,3,3-trifluoro-2-(4-fluoroanilino)propionate	26.484	0.38
35	Dodecahydropropyrido [1,2-b]isoquinolin-6-one	26.528	0.49
36	1-methyl-4-phenyl -5-thioxo-1,2,4-triazo[idin3-one	26.595	0.58
37	Ethyl 2-(2-chloroacetimido)-3,3,3-trifluoro-2-(4-fluoronilino)propionate	26.784	3.63
38	1H-Isonidole-1,3(2H)-dione,2-buty1-4,5,6,7-tetrahydro-	27.017	4.26
39	Indole-2-one,2,3-dihydro-N-hydroxy-4-methoxy-3,3-dimethyl-	27.162	4.36
40	Dodecahydropyrido(1,2-b)isoquinoli-n-6-one	27.284	1.68
41	Ethly 2-(2- chloroacetamido-3,3,3-trifluoro-2-(3-fluoroanilino)propionate	27.817	0.55
42	1,2-Bis(trimethylsilyl)benzene	29.073	5.81
43	Octatriacontyl pentafluoropropionate	29.817	2.97
44	2′-Hydroxypropiophenone,TMS derivative	30.184	2.33

### 3.4 Chemical component of goat horn oil extract

**Fig. 2:**
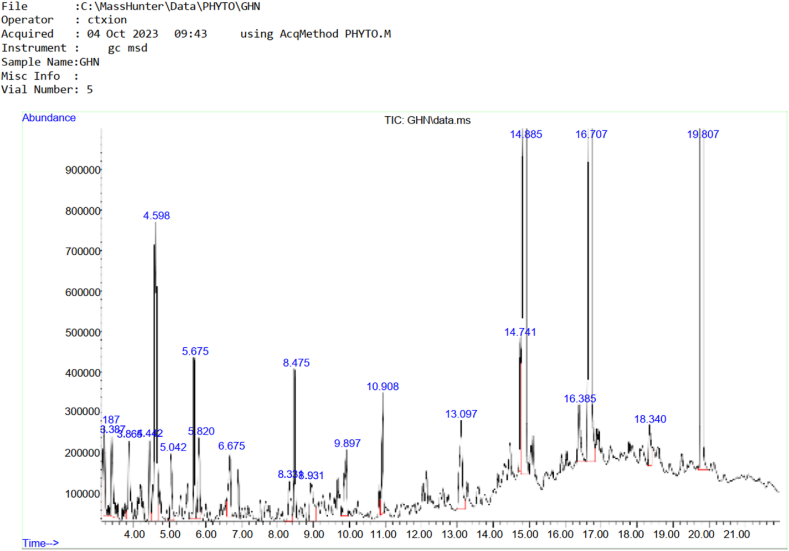
Chromatogram of the chemical compounds present in Goat Horn.

Table 4 showed 21 biochemical compounds present in goat horn oil extract while Fig. 2 showed Chromatogram of the chemical compounds present in goat horn. The chemical compound with the highest area percent among the identified compounds was Diisooctyl phthalate with area percent of 27.75, followed by n-Hexadecanoic acid with 19.94 area percent and Octadecanoic acid with 17.85 area percent. Other biochemicals present were 2H-Benzotriazole, 2-ethyl (7.44), Octadecane (2.70), Tetradecane (2.13), and Dibutyl phthalate (2.03). Chemical compounds with low area % present in the goat horn oil extract include 2-Piperidinone, N-[4-bromo-n-butyl]- with 0.88 area percent and Dichloroxylenol with area percent of 0.98.

**Table 4:** Chemical compounds present in goat horn oil extract.

**S/N**	Compound	Retention Time	Area %
1	Cyclotetrasiloxane, octamethyl-	3.187	1.19
2	Benzene,1,2,3-trimethyl-	3.387	1.62
3	Decane,3,6-dimethyl-	3.865	1.62
4	Undecane	4.442	1.29
5	2H-Benzotriazole, 2-ethyl	4.598	7.44
6	4H-Pyran-4-one, 2,3-dihydro-3, 5-dihydroxy-6-methyl	5.042	1.26
7	(1R, 3R, 4R, 5S)-1-Isopropyl-4-methylbicyclo [3.1.0] hexan-3-yl acetate-rel-	5.675	2.24
8	Dodecane	5.820	1.33
9	Benzeneacetic acid	6.675	1.48
10	Dichloroxylenol	8.331	0.98
11	Tetradecane	8.475	2.13
12	Benzeneethanol, 4-hydroxy-	8.931	1.48
13	2,4-Di-tert-butylphenol	9.897	1.63
14	Hexadecane	10.908	1.69
15	Octadecane	13.097	2.70
16	Dibutyl phthalate	14.741	2.03
17	n-Hexadecanoic acid	14.885	19.94
18	Cyclopentadecane	16.385	1.46
19	Octadecanoic acid	16.707	17.85
20	2-Piperidinone, N-[4-bromo-n-butyl]-	18.340	0.88
21	Diisooctyl phthalate	19.807	27.75

### 3.5 Chemical component of cow horn oil extract

**Fig. 3:**
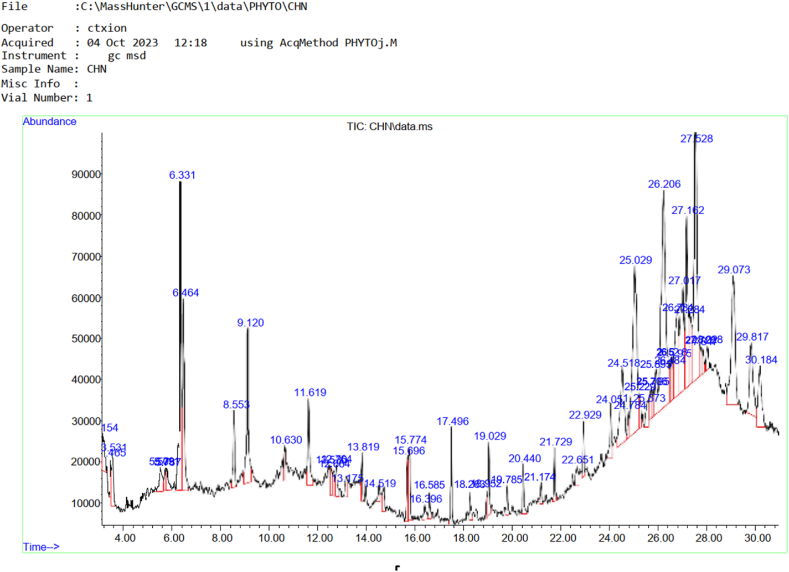
Chromatogram of the chemical compounds present in Cow Horn.

Table 5 showed 18 biochemicals compounds present in goat horn oil extract while Fig. 3 showed Chromatogram of the chemical compounds present in cow horn. The chemical compound with the highest area percent among the identified compounds was Diisooctyl phthalate with area percent of 21.25, followed by n-Hexadecanoic acid with 17.72 area percent and Octadecanoic acid with 15.33 area percent. Other biochemicals present were 2H-Benzotriazole, 2-ethyl (8.42), Octadecane (3.71), Tetradecane (2.44), and Dibutyl phthalate (2.87). Chemical compound with low area % present in the goat horn oil extract include 2-Piperidinone, N-[4-bromo-n-butyl]- with 0.88 area percent and Dichloroxylenol with area percent of 0.98.

**Table 5:** Chemical compounds present in cow horn oil extract.

**S/N**	Compound	Retention Time	Area %
1	Cyclotetrasiloxane, octamethyl-	3.187	1.19
2	Benzene,1,2,3-trimethyl-	3.387	1.62
3	Decane,3,6-dimethyl-	3.865	1.62
4	Undecane	4.442	1.29
5	2H-Benzotriazole, 2-ethyl	3.899	8.42
6	Dodecane	5.820	1.33
7	Benzeneacetic acid	6.675	1.48
8	Dichloroxylenol	8.672	0.84
9	Tetradecane	8.475	2.13
10	Benzeneethanol, 4-hydroxy-	8.931	1.48
11	Hexadecane	10.908	1.69
12	Octadecane	13.097	2.70
13	Dibutyl phthalate	14.741	2.03
14	n-Hexadecanoic acid	13.862	17.32
15	Cyclopentadecane	16.385	1.46
16	Octadecanoic acid	16.707	15.33
17	2-Piperidinone, N-[4-bromo-n-butyl]-	17.367	0.76
18	Diisooctyl phthalate	18.807	21.25

### 3.6 Chemical component of goat hoof oil

**Fig. 4:**
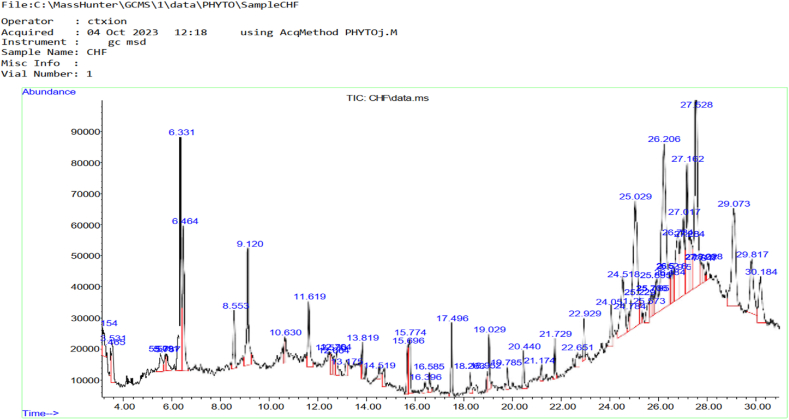
Chromatogram of the chemical compounds present in goat hoof.

The chemical compounds found in cow hoof oil extract is presented in Table 6 and Chromatogram of the chemical compounds present in goat hoof is also presented in Fig. 4. Total number of 46 bioactive compounds were identified from the oil sample. The compound with the highest area percentage among the identified compounds was 1,2-Benzenediol,3,5-bis(1,1-dimethylethyl) with area percent of 10.79, this was followed by Undec-10-ynoic acid tetradecyl ester with 7.99 area percent and 1,2-Bis(trimethylsilyl)benzene with area percent of 5.81. Other notable compounds present include Cyclotrisiloxane (tert-butyldimethysilyloxy and terimethsilyl), 3-Hydromandelic acid,1H-Isonidole-1,3(2H)-dione,2-buty1-4,5,6,7-tetrahydro-, Indole-2-one,2,3-dihydro-N-hydroxy-4-methoxy-3,3-dimethyl-, and Arsenous acid tris (trimethylsilyl)ester.

**Table 6:** Chemical compounds present in goat hoof oil extract.

**S/N**	Compound Detected	Retention Time	Area %
1	Cyclotrisiloxane, Hexamethyl-1,2-bezisothiazol-3-amine (tert-butyldimethysilyloxy)	3.154	0.83
2	Cyclotrisiloxane, Hexamethyl-1,2-bezisothiazol-3-amine (terimethsilyl)	3.465	0.46
3	9H-Fluorene-2-carboxylicacid	3.531	1.06
4	Silicic acid	5.731	3.26
5	3-Hydromandelic acid	8.553	1.32
6	Cyclopentasiloxane, decamethl-cylopentasiloxane	9.120	3.22
7	1,3,5,7,9-Pentaethylcyclopentasiloxane	10.630	0.46
8	Cyclohexasiloxane, dodecamethyl-cyclohexasiloxane	11.619	1.86
9	Pyrido[-2,3-d]pyrimidine,4-phenyl-	12.530	0.52
10	Cyclotrisiloxane	12.764	0.60
11	Carvacol	13.175	0.57
12	Cyclopentasiloxane	13.819	0.60
13	5,5′-Di(ethoxycarbonyl)-3,3′-dimethyl-4,4′-dipropyl-2,2′-dipyrrylmethane	14.519	0.33
14	Cyclopentasiloxane, decamethyl-3,4-dihydroxyphenylglycol	15.696	0.99
15	Epineprine,(.beta.)-,3TMS derivative	15.774	1.47
16	Octasiloxane,1,1,3,3,5,5,7,7,9,9,11,11,13,13,15,15- hexadecamethyl-	17.496	1.27
17	1H-Indole-2-carboxylic acid,6-(4-ethoxyphenyl)-3-methyl-4-oxo-4,5,6,7-tetrahydro-,Isopropyl ester	18.263	1.43
18	Tris(tert-butyldimethylsilyloxy)ar sane	18.952	0.34
19	Heptasiloxane,1,1,3,3,5,5,7,7,9,9,11,11,13,13-tetradecamethyl-	19.029	1.03
20	2,4-Dihydroxybenzoic acid, 3TMS derivative	20.440	0.70
21	Arsenous acid, tris(trimethylsilyl)ester	21.174	0.36
22	4,7,7-Trimethylbicyclo[2.2.1]heptan-2-one 0-allylxime	22.651	0.57
23	1,2,5-Oxadiazole-3-amine,4-(4-methoxyphenoxy)-	22.929	0.69
24	1H-Indole-2-carboxylic acid	24.05	1.02
25	1,2-Benzenediol,3,5-bis(1,1-dimethylethyl)-	24.518	3.26
26	4H-1,2,4-triazole-3,5-diamine,N3-($-fluorophenyl)-N5-methyl-	24.784	0.50
27	Undec-10-ynoic acid, tetradecyl ester	25.029	7.99
28	2-methyl-z,z-3,13-octadecadienol	25.229	0.54
29	Pyrazolo[5,1-c][1,2,4]triazine-3,8-dicarboxylic acid,4-amino-,diethyl ester	25.573	0.75
30	11,13-Dimethyl-12-tetradecen-1-ol acetate	25.706	0.65
31	2-Myristynoyl-glycinamide	25.795	0.49
32	1H-Isonidole-1,3(2H)-dione,2-buty 1–4,5,6,tetrahydro-	25.895	1.19
33	1,2-Benzenediol,3,5-bis(1,1-dimethylethyl)-	26.206	10.79
34	Ethyl 2(-2-chloroacetamido)-3,3,3-trifluoro-2-(4-fluoroanilino)propionate	26.484	0.38
35	Dodecahydropropyrido [1,2-b]isoquinolin-6-one	26.528	0.49
36	1-methyl-4-phenyl -5-thioxo-1,2,4-triazo[idin3-one	26.595	0.58
37	Ethyl 2-(2-chloroacetimido)-3,3,3-trifluoro-2-(4-fluoronilino)propionate	26.784	3.63
38	1H-Isonidole-1,3(2H)-dione,2-buty1-4,5,6,7-tetrahydro-	27.017	4.26
39	Indole-2-one,2,3-dihydro-N-hydroxy-4-methoxy-3,3-dimethyl-	27.162	4.36
40	Dodecahydropyrido(1,2-b)isoquinoli-n-6-one	27.284	1.68
41	Ethly 2-(2- chloroacetamido-3,3,3-trifluoro-2-(3-fluoroanilino)propionate	27.817	0.55
42	1,2-Bis(trimethylsilyl)benzene	29.073	5.81
43	Octatriacontyl pentafluoropropionate	29.817	2.97
44	2′-Hydroxypropiophenone,TMS derivative	30.184	2.33
45	9-Octadecenoic acid (Z)-, methyl ester	30.264	2.87
46	Eicosyl isopropyl ether, Isopropyl octadecyl ether, Sulfurous acid, butyl octadecyl ester	31.211	3.44

### 3.7 Insecticidal properties of oil extracted from hoof and horn of cow and goat against adult *Rhyzopertha dominica*

The insecticidal activities of oil extracted from hoof and horn of cow and goat against adult *R. dominica* is presented in Table 7. From the Table, results showed a dose-and-time dependent mortality. The highest mortality of adult *R. dominica* was observed from the effect of goat horn oil. There is a significant difference (p < 0.05) in toxicity effect of 0.1 ml of goat horn oil compared to the same concentration of goat hoof, cow hoof and cow horn oils after day 1 of application. 0.1 ml of goat horn oil evoked 10 % mortality of adult *R. dominica*, while 0.1 ml of goat hoof oil, cow hoof and cow horn oils have no toxicity effect on the insect. There is no significant difference (p > 0.05) when comparing 0.1 ml goat hoof, cow hoof and horn oil with the control after day 1 of application. The effect of 0.1 ml of goat horn oil, 0.3 ml of cow horn oil and 0.4 ml of goat hoof oil were not different significantly (p > 0.05) as 10 % mortality of *R. dominica* was observed in all the treatments after day 1 of application. The highest mortality (56.67 %) of *R. dominica* after day 1 of post treatment was observed from the toxicity effect of 0.5 ml goat horn oil, this is different significantly from the effect of goat hoof, cow hoof and horn oil where 16.67 %, 40 % and 26.67 % mortality were recorded respectively.

**Table 7:** Toxicity of Animals Hoof and Horn oils on Mortality of Adult *Rhyzopertha dominica* on Wheat Grains.

Animal Oil	Conc. (ml)	%Mortality				
		Day 1	Day 2	Day 3	Day 4	Day 5
CHN	0.1	0.00 ± 0.00a	3.33 ± 3.33ab	10.00 ± 0.00b	13.33 ± 3.33bc	16.67 ± 3.33b
CHF		0.00 ± 0.00a	10.00 ± 0.00bc	13.33 ± 3.33bc	16.67 ± 3.33bc	20.00 ± 0.00bc
GHN		10.00 ± 0.00b	20.00 ± 0.00de	26.67 ± 3.33de	30.00 ± 0.00d	36.67 ± 3.33d
GHF		0.00 ± 0.00a	0.00 ± 0.00a	3.33 ± 3.33a	10.00 ± 0.00b	13.33 ± 3.33b
CHN	0.2	3.33 ± 3.33a	6.67 ± 3.33abc	13.33 ± 3.33bc	20.00 ± 0.00c	26.67 ± 3.33c
CHF		6.67 ± 3.33ab	13.33 ± 3.33cd	20.00 ± 0.00cd	30.00 ± 0.00d	36.67 ± 3.33d
GHN		20.00 ± 0.00c	26.67 ± 3.33ef	36.67 ± 3.33fg	43.33 ± 3.33ef	50.00 ± 0.00e
GHF		0.00 ± 0.00a	3.33 ± 3.33ab	13.33 ± 3.33bc	16.67 ± 3.33bc	20.00 ± 5.77bc
CHN	0.3	10.00 ± 0.00b	20.00 ± 0.00de	30.00 ± 0.00ef	40.00 ± 0.00e	50.00 ± 0.00e
CHF		20.00 ± 0.00c	30.00 ± 0.00fg	40.00 ± 0.00gh	46.67 ± 3.33ef	56.67 ± 3.33ef
GHN		30.00 ± 0.00de	40.00 ± 0.00hi	53.33 ± 3.33i	60.00 ± 0.00g	70.00 ± 0.00g
GHF		3.33 ± 3.33a	10.00 ± 0.00bc	20.00 ± 0.00cd	30.00 ± 0.00d	36.67 ± 3.33d
CHN	0.4	20.00 ± 0.00c	30.00 ± 0.00fg	40.00 ± 0.00gh	50.00 ± 0.00f	60.00 ± 0.00f
CHF		33.33 ± 3.33e	43.33 ± 3.33i	50.00 ± 0.00i	56.67 ± 0.00g	70.00 ± 0.00g
GHN		43.33 ± 3.33f	53.33 ± 3.33j	63.33 ± 3.33j	73.33 ± 3.33h	90.00 ± 0.00h
GHF		10.00 ± 0.00b	20.00 ± 0.00de	30.00 ± 0.00ef	40.00 ± 0.00e	50.00 ± 0.00e
CHN	0.5	26.67 ± 3.33d	36.67 ± 3.33gh	46.67 ± 3.33hi	60.00 ± 0.00g	70.00 ± 0.00g
CHF		40.00 ± 0.00f	50.00 ± 0.00j	60.00 ± 0.00j	76.67 ± 3.33h	86.67 ± 3.33h
GHN		56.67 ± 3.33g	66.67 ± 3.33k	76.67 ± 3.33k	86.67 ± 3.33i	100.00 ± 0.00i
GHF		16.67 ± 3.33c	30.00 ± 0.00fg	40.00 ± 0.00gh	50.00 ± 0.00f	56.67 ± 3.33ef
Control	0.0	0.00 ± 0.00a	0.00 ± 0.00a	0.00 ± 0.00a	0.00 ± 0.00a	0.00 ± 0.00a

Mean with the same letter in superscript along column are not significantly different from one another (*p* > 0.05) using Duncan Multiple Range Test.

Keys: CHN: cow horn; CHF: cow hoof; GHN: goat horn; GHF goat hoof.

The toxicity of the treatments increased as the period of exposure increased, however, no *R. dominica* mortality was recorded after day 2 of applying 0.1 ml of goat hoof oil. There is no significant difference (p > 0.05) in toxicity of 0.1 ml and 0.2 ml of goat horn oil (20 % and 26.67 % mortality were observed respectively), 0.3 ml of cow horn oil (20 % mortality was observed), and 0.4 ml of goat hoof oil (20 % mortality was observed) after day 2 post treatment. There is no significant difference (p > 0.05) in insecticidal activities of 0.4 ml of goat horn oil and 0.5 ml of cow hoof oil after day 2 of application, with 53.33 % and 50 % mortality of *R. dominica* recorded respectively. After day 3, 4 and 5 post treatment, the effect of 0.5 ml goat horn oil was the highest, with 76.67 %, 86.67 % and 100 % mortality of *R. dominica* observed, respectively. Concentration of 0.5 ml from goat hoof oil evoked the least mortality of *R. dominica* when compared with the same concentration of goat horn oil, cow hoof and horn oils. There is no significant difference (p > 0.05) in the adulticidal effect of 0.3 ml of goat horn oil, 0.4 of cow hoof oil and 0.5 ml of cow horn oil, since 70 % mortality of adult *R. dominica* was recorded.

### 3.8 Estimation of required lethal concentration (LC_50_ and LC_90_) of animal oil against *Rhyzopertha dominica*

Table 8 shows the lethal concentration of oil extracted from horn and hoof of cow and goat that is required to evoke 50 % and 90 % mortality of adult *R. dominica* on wheat grains. The estimated effectiveness of the animal oil is time-dependent. The concentration of animal oil extracted from cow horn, cow hoof, goat horn, goat hoof required to evoke 50 % mortality of *R. dominica* within the period of 24 h post treatment application were 0.77 ml, 0.57 ml, 0.48 ml, and 0.87 ml respectively, while the amount required for 90 % mortality were 3.04 ml, 2.49 ml, 2.08 ml, and 2.75 ml respectively. For day 3 after application, 0.59 ml and 2.0 ml of cow horn was estimated to cause 50 % and 90 % mortality of adult *R. dominica*, this was lightly higher compared with cow hoof oil where 0.41 ml and 1.44 ml were required respectively. The amount of goat horn and hoof oils required to evoke 50 % and 90 % mortality of adult *R. dominica* differ; 0.25 ml and 1.21 ml of goat horn oil is needed to evoke 50 % and 90 % mortality respectively, while 0.68 ml and 2.04 ml of goat hoof oil is needed to evoke same mortality rate after day 3 of application. At day 5, the amount of goat horn oil (0.16 ml and 0.53 ml) estimated to achieve 50 % and 90 % mortality of *R. dominica* were smaller compared to goat hoof oil (0.43 ml and 1.76 ml), cow horn oil (0.31 ml and 1.19 ml) and cow hoof oil (0.23 ml and 0.72 ml)

**Table 8:** Estimated lethal concentration (LC_50_ and LC_90_) of animal oil on *Rhyzopertha dominica*.

Animal Oil	Exposure Period	Intercept± S.E.	Slope± S.D.	*R*^2^	LC_50_ (LCL - UCL)	LC_90_ (LCL -UCL)	*p* value
CHN	Day 1	5.35 ± 0.08	3.12 ± 0.32	0.99	0.77 (0.54–1.11)	3.04 (1.88–4.93)	0.82
	Day 2	5.31 ± 0.10	2.26 ± 0.44	0.96	0.73 (0.47–1.12)	2.74 (1.78–4.21)	0.68
	Day 3	5.42 ± 0.11	1.83 ± 0.55	0.92	0.59 (0.37–0.96)	2.00 (1.39–2.88)	0.53
	Day 4	5.79 ± 0.09	2.01 ± 0.49	0.95	0.41 (0.27–0.62)	1.78 (1.17–2.71)	0.62
	Day 5	6.12 ± 0.09	2.19 ± 0.46	0.96	0.31 (0.21–0.45)	1.19 (0.81–1.75)	0.69
CHF	Day 1	5.78 ± 0.07	3.20 ± 0.31	0.99	0.57 (0.42–0.78)	2.49 (1.59–3.91)	0.57
	Day 2	5.53 ± 0.10	1.96 ± 0.51	0.92	0.54 (0.34–0.85)	1.78 (1.17–2.71	0.44
	Day 3	5.79 ± 0.09	2.02 ± 0.50	0.95	0.41 (0.27–0.62)	1.44 (1.05–1.96)	0.62
	Day 4	6.19 ± 0.08	2.28 ± 0.44	0.94	0.30 (0.21–0.42)	1.11 (0.77–1.61)	0.72
	Day 5	6.68 ± 0.07	2.66 ± 0.38	0.95	0.23 (0.17–0.32)	0.72 (0.52–0.99)	0.69
GHN	Day 1	5.65 ± 0.09	2.01 ± 0.49	0.97	0.48 (0.31–0.74)	2.08 (1.35–3.19)	0.80
	Day 2	5.80 ± 0.10	1.78 ± 0.56	0.91	0.36 (0.22–0.57)	1.89 (1.19–3.02)	0.57
	Day 3	6.14 ± 0.10	1.88 ± 0.53	0.94	0.25 (0.16–0.39)	1.21 (0.78–1.88)	0.75
	Day 4	6.57 ± 0.08	2.24 ± 0.45	0.93	0.19 (0.14–0.29)	0.76 (0.52–1.11)	0.70
	Day 5	7.02 ± 0.08	2.52 ± 0.39	0.87	0.16 (0.11–0.23)	0.53 (0.37–0.75)	0.41
GHF	Day 1	5.24 ± 0.08	3.93 ± 0.25	0.99	0.87 (0.61–1.25)	2.75 (1.71–4.41)	0.41
	Day 2	5.47 ± 0.08	3.31 ± 0.30	0.99	0.72 (0.51–1.02)	2.59 (1.69–4.00)	0.89
	Day 3	5.37 ± 0.09	2.19 ± 0.46	0.99	0.68 (0.44–1.04)	2.04 (1.31–3.18)	0.97
	Day 4	5.48 ± 0.11	1.85 ± 0.54	0.96	0.55 (0.35–0.89)	1.84 (1.28–2.64)	0.79
	Day 5	5.69 ± 0.10	1.92 ± 0.52	0.95	0.43 (0.28–0.68)	1.76 (1.25–2.48)	0.67

Keys: CHN: cow horn; CHF: cow hoof; GHN: goat horn; GHF goat hoof.

Note: R^2^ = Statistical measure of mortality proportion in regression model.

S. E. = Standard error.

S. D. = Standard deviation.

LD_50_ = Lethal dosage at which 50 % population response.

LD_90_ = Lethal dosage at which 90 % population response.

LCL = Lower confidence limit.

UCL = Upper confidence limit.

p value = Chi -square (*X*^2^) Significant.

### 3.9 Effect of animal oil extract on number of *Rhyzopertha dominica* adult emergence

Table 9 showed the effectiveness of animal oil extract against *R. dominica* adult emergency. The highest concentration of the animal oil extracts gave the lowest adult emergency. Lowest mean of emergence of adult *R. dominica* was observed from the application of 0.5 ml of goat horn oil extract and cow hoof oil extract, with 1.33 and 2.67 mean adult emergency recorded respectively. The effectiveness of 0.4 ml of goat oil extract and 0.5 ml of cow hoof oil extract were the same and not statistically significant (p > 0.05), 2.67 mean adult emergency was observed from both concentrations of the extracts. The effect of 0.3 and 0.4 ml of goat horn oil extract (4.33 and 2.67 was observed respectively) was not different significantly from the effect of 0.4 and 0.5 ml of cow hoof oil extract (4.33 and 2.67 was observed respectively). The highest (19.00) number of adult emergence was observed from the application of 0.1 ml of goat hoof oil; though this value was significantly different (p < 0.05) from the control which produced 39 adult emergence.

**Table 9:** Effect of animal oils on adult emergence of *Rhyzopertha dominica*.

Animal oil	Conc. (ml)	No of Adult Emergence
CHN	0.1	15.67 ± 0.88^k^
CHF		12.33 ± 0.88^j^
GHN		9.00 ± 0.58^fgh^
GHF		19.00 ± 0.58^l^
CHN	0.2	12.00 ± 0.58^ij^
CHF		10.33 ± 0.33^hi^
GHN		6.67 ± 0.33^de^
GHF		17.00 ± 0.58^k^
CHN	0.3	8.00 ± 0.58^efg^
CHF		6.33 ± 0.33^de^
GHN		4.33 ± 0.33^bc^
GHF		12.00 ± 0.58^ij^
CHN	0.4	6.00 ± 0.58^cd^
CHF		4.33 ± 0.33^bc^
GHN		2.67 ± 0.88^ab^
GHF		9.67 ± 0.33^gh^
CHN	0.5	4.00 ± 0.58^b^
CHF		2.67 ± 0.33^ab^
GHN		1.33 ± 0.33^a^
GHF		7.33 ± 0.33^def^
Control	0.0	39.00 ± 1.16^m^

Mean with the same letter in superscript along column are not significantly different from one another (*p* > 0.05) using Duncan Multiple Range Test’.

Keys: CHN: cow horn; CHF: cow hoof; GHN: goat horn; GHF goat hoof.

### 3.10 Pearson correlation between proximate parameters in animal extracts and adult emergence of *Rhyzopertha dominica*

**Fig. 5:**
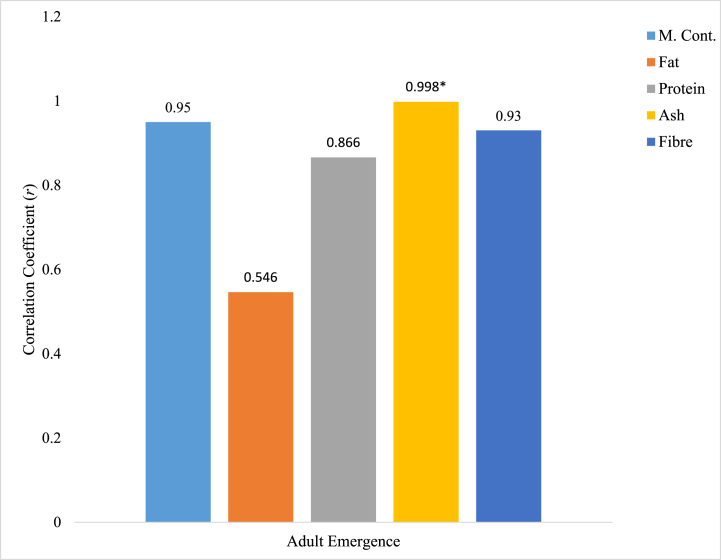
Correlation relationship between proximate parameters of cow horn crude extracts and *R. dominica* adult emergence. ∗ Correlation is significant at the 0.05 level (2-tailed).

**Fig. 6:**
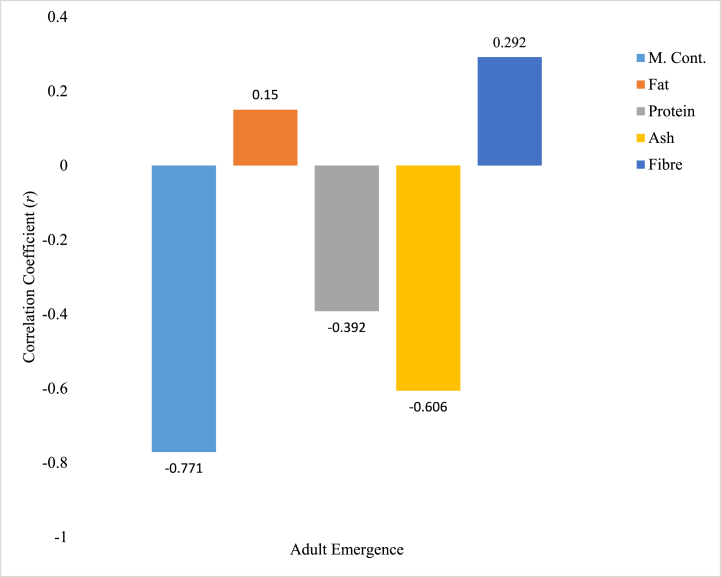
Correlation relationship between proximate parameters of cow hooves crude extracts and *R. dominica* adult emergence.

Relationship between proximate parameters of cow (horn and hoof) crude extracts and number adult emergence of *R. dominica* is shown in Fig. 5, Fig. 6. All the proximate parameters quantified in cow horn crude extract were positively related with the level of adult *R. dominica* emergence; the correlation between moisture (0.950), protein (0.866), Ash (0.998), and fibre (0.930) content in cow horn crude extract were strongly and positively related with number of adult *R. dominica* observed. However, only Ash content of cow horn crude extract was positively significant (p < 0.05) with number of *R. dominica* adult emergence. The fat content of cow horn crude extract was positively and moderately related to the number of *R. dominica* adult emergence (Fig. 5). In Fig. 6, the correlation between moisture (−0.771), protein (−0.392), and Ash (−0.606) contents of cow hoof crude extract was negatively related, while fat (0.150) and fibre (0.292) content of the extract were weak and positively related.

**Fig. 7:**
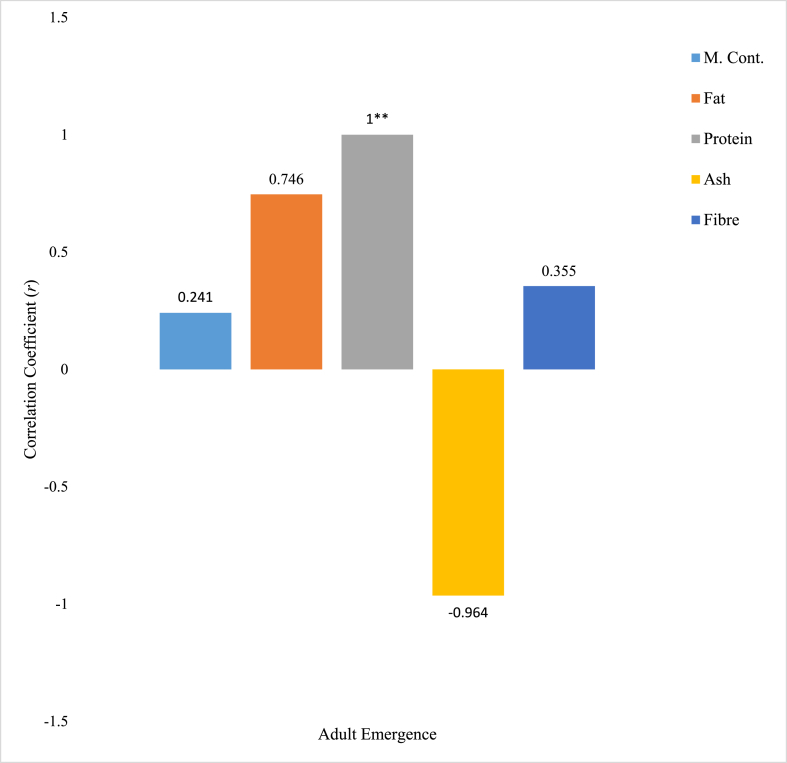
Correlation relationship between proximate parameters of goat horn crude extracts and *R. dominica* adult emergence. ∗∗ Correlation is significant at the 0.01 level (2-tailed).

**Fig. 8:**
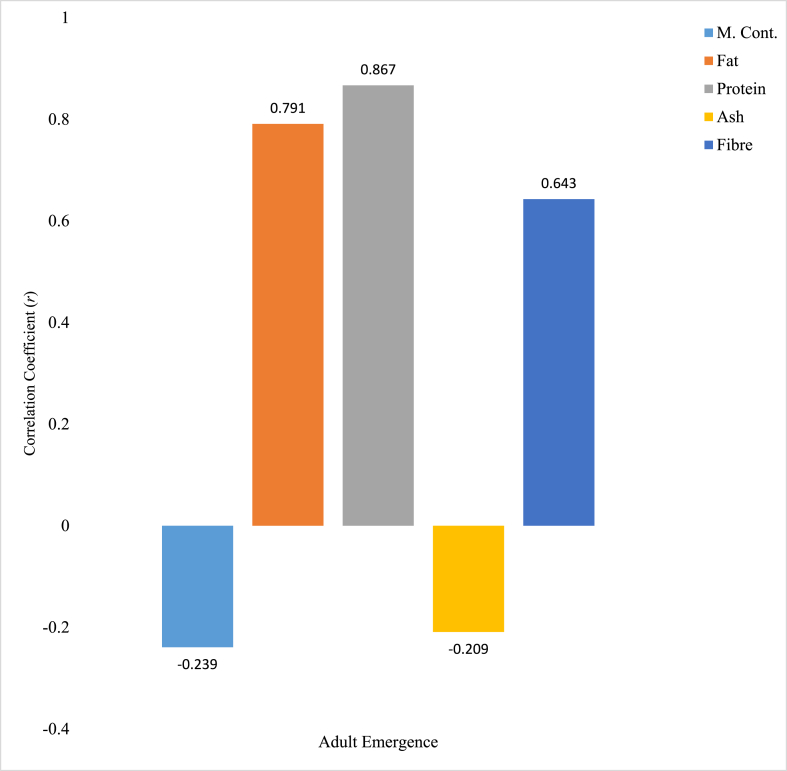
Correlation relationship between proximate parameters of cow hooves crude extracts and *R. dominica* adult emergence.

Correlation between the *R. dominica* adult emergence and goat horn crude extract proximate parameters was given in Fig. 7. Out of all the proximate parameters considered, only Ash content of the extract was found to have strong negative relationship with the number of *R. dominica* adult emergence with −0.964 correlation coefficient (*r*). Protein content of the goat horn crude extract was positively and significantly (p < 0.05) correlated with *R. dominica* adult emergence with 1.000 correlation coefficient. Fat content of the extract was positively and moderately correlated with *R. dominica* adult emergence with correlation coefficient (*r*) of 0.746. Moisture (0.241) and fibre (0.355) contents of the extract have a positive and weak correlation with *R. dominica* adult emergence. Fig. 8 shows the correlation between *R. dominica* adult emergence and goat hoof crude extract proximate parameters. Moisture (−0.239) and Ash (−0.209) contents of the extract were negatively and weakly correlated with *R. dominica* adult emergence. Fat and fibre contents of the extract were positively and moderately correlated with *R. dominica* adult emergence, with 0.791 and 0.643 correlation coefficient calculated respectively. Only protein content of the extract is strongly and positively related to *R. dominica* adult emergence with 0.867 correlation coefficient.

## 4 Discussion

Animal horn and hoof are important structure in animal body that enhances their functional ability. Products from animal horn and hoof have been reportedly used in cosmetic industries for the production of shampoos, moisturizers, masques and conditioner [28,29]. More so, in agricultural industries majorly for fertilizer [30] and animal meal [16]. In another study, Goni et al. [30] reported the use of animal bone, hoof and horn materials as component of fire extinguisher fluid. However, there is limited information on the use of animal horn and hoof products in the control of store pest products. The present study has revealed the potency of goat and cow horn and hoof extracts in the management of lesser grain borer, *R. dominica* a major field-to-store insect pest infesting dried wheat grains. It was observed that the insecticidal potency of the animal horn and hoof extracts was dosage- and time-dependent i.e., the potency of the animal extract increased as the concentration and period of exposure increases. Findings from the study showed that goat horn extract was more toxic against *R. dominica*. These findings were consistent with the report of Ileke and Ojomo [16] who reported the insecticidal potency of animal bones biochar for the management of rice weevil *S. oryzae*. In another study, Nwosu et al. [20] gave account of animal bone charcoal insecticidal efficacy as an alternative to synthetic chemical in the management of maize weevil, *S. zeamais*.

The insecticidal effectiveness of hoof and horn extracts of cow and goat was found to be dosage-dependent. The mortality of the studied insect increased as the hoof and horn extract dosage increased. The present study also revealed the protectability of the animal extract by inhibiting oviposition of the insect which led to reduction of adult emergency as the dosage of the extract increases. Tajchman et al. [31] reported that animals accumulate toxic substances in their bones, hooves and horn. These toxic substances are not harmful to the animal if at lower concentration; this bioaccumulation is as result of different food materials the animals foraged on over the years. These toxic substances in the animal materials are major suspect of insecticidal activities.

Gas chromatography mass-spectrophotometry analysis revealed that the understudy animal horn and hoof possess bioactive chemical compounds which their uses are not well documented or publicly available. However, some of these chemical compounds have been used in the pharmaceuticals and agrochemicals companies. One of the chemical compounds identified in cow hoof, 1H-Isonidole1,3(2H)-dione, 2-buty 1–4,5,6, tetrahydro a derivate of isonidole, is a heterocyclic compound whose function and application has been documented in the development of agrochemical such as fungicides and herbicides. In a review reported by Lamberth [31], the significance of heterocyclic compounds in crop protection was documented as fungicides, herbicides, insecticides, and pro-pesticides. In another study, adulticidal, larvicidal, ovicidal, repellent, and oviposition-deterrent activities of 1,2-Benzenediol,3,5-bis(1,1-dimethylethyl)-phenol was reported against the spider mite [32] Cyclotetrasiloxane also known as octamethylcyclotetrasiloxane, a chemical compound common to cow hoof and goat horn extracts which are the most active animal extract against the studied pest has been described as primary component of silicone polymers. In addition, this material is utilized as a solvent and emollient in a range of personal care items, such as skin creams, hair care products, deodorants, and makeup [[33], [34], [35]]. Furthermore, Octamethylcyclotetrasiloxane is utilized in industrial products such as lubricants, cleaning supplies, sealants, adhesives, waxes, polishes, and coatings; it also functions as a defoamer and surfactant in some pesticide applications [36].

The current study revealed the trace elements and proximate content present in cow and goat hoof and horn extracts. The present study shown that the hoof and horn extracts from cow and goat contain different percentage of trace elements such as Na, K, Ca, Mn, Mg, Cu, Fe, Zn, and P. Composition of these elements in biological materials such as blood, hoof and horn of animal are important indicator of environmental pollution [37]. Biological monitoring of trace elements and chemical compounds in animal biomaterials is a good tool for the investigation of potential bioaccumulation in urbanized and industrialized geographical area [38]. Furthermore, monitoring the mineral composition of animal biomaterials gives more details of their health and what they are exposed to in the environment most especially what the animals feed on. All the trace metals reported from the cow and goat hoof and horn are essential for the good health of the animal. The findings were not different from the report of Langova et al. [39] who reported same essential trace metals in cattle.

The proximate composition of the animal hoof and horn showed different percentage composition of moisture content, fat, protein, ash and fibre. The percentage of proximate composition parameters reported in the current study is different from the value reported in the liver, kidney and intestine of the animal by Bristone et al. [40]. Langova et al. [39] reported that the healthy status of animal hoof and horn depend on the nutritional content in animal feeds. According to Langova et al. [39], animal hoof and horn disease prevalence has been linked to poor nutrition, which is one of the fundamental preventive factors influencing the development and quality of the hoof and horn in animals. The composition of the feed rations an animal consumes influences the strength and shape of its hoof and horn.

## 5 Conclusion

The crude extract of the animal horn and hoof understudied was found to contain bioactive compounds which define their toxic activities against *R. dominica*. Also, these bioactive compounds have been proven for their efficacy in pesticide, fertilizer, cosmetic, and fire extinguisher production. Thus, this study did not only identify the active component of goat and cow horns and hoofs; but also, add more knowledge to the body of literature. Findings from this study have shown that animal horn and hoof extracts are valuable animal materials for the control and management of lesser grain borer, *R. dominica*.

## CRediT authorship contribution statement

**Kayode David Ileke:** Writing – review & editing, Supervision, Project administration, Methodology, Conceptualization. **Catherine Olukemi Adeniran:** Writing – original draft, Investigation, Conceptualization.

## Ethics approval and consent to participate

Not applicable.

## Consent for publication

Not applicable.

## Data availability statement

Research data had been included in supporting information online.

## Funding

No research fund was received.

## Declaration of competing interest

The authors declare that they have no known competing financial interests or personal relationships that could have appeared to influence the work reported in this paper.

## Abbreviations

FUTA: Federal University of Technology, Akure
ANOVA: Analysis of variance
SE: Standard Error
LD: Lethal Dose
LC: Lethal Concentration
CHN: cow horn
CHF: cow hoof
GHN: goat horn
GHF: goat hoof
